# The Importance of Functional Quality in Patient Satisfaction: Cosmetic Injectable Patient Experience Exploratory Study—Part 2

**DOI:** 10.1093/asjof/ojac044

**Published:** 2022-05-08

**Authors:** Cara B McDonald, Izolda Heydenrych

**Affiliations:** Department of Dermatology, St Vincent's Hospital, Melbourne

## Abstract

**Background:**

Quality assessment comprises 2 distinct forms: technical quality (TQ) and functional quality (FQ). On the one hand, TQ describes accuracy and excellence, the degree to which procedures are done “correctly.” On the other hand, FQ is the way services are delivered and represents how the customer perceives and experiences the treatment or service.

**Objectives:**

To determine the relative importance of functional quality factors in the care of cosmetic injectable patients and return patronage.

**Methods:**

The Cosmetic Injectable Patient Experience Exploratory Study (CIPEES) survey assessed reasons for return patronage to a specific cosmetic injector and the correlation between satisfaction with cosmetic results (patient assessment of TQ) and respondents’ trust level in their practitioner, a marker for FQ.

**Results:**

The CIPEES survey collected 1488 responses across 75 countries, with 66% of participants completing all 15 questions. The respondents were 95.6% female and 4.4% male, with ages ranging from 18 years to >65 years old (median 33 years old). The number one ranked reason for returning to a previous cosmetic injector (return patronage) was “Trust in my practitioner’s action and ability,” closely followed by “Cosmetic result/outcome from the previous treatment/s.” Respondents’ level of satisfaction with their cosmetic results also correlated highly with trust in their practitioners.

**Conclusions:**

In order to maximize patient satisfaction and return patronage, healthcare practitioners should focus on improving FQ care and value it at least as high as TQ in the delivery of cosmetic injectable treatments.

Patient satisfaction has long been considered an essential measure of success in the field of medicine and healthcare delivery. Yet, the measurement and reporting of patient satisfaction remain elusive and complex. Tools for measuring patient satisfaction in healthcare are available but tend to be specific to particular fields rather than universal. Many assessments focus on technical outcomes and return to normal function, with metrics around pain, complications, recovery time, and value for money.

With regard to patient satisfaction within the aesthetic medicine literature, most publications focus on improvement in quality of life following aesthetic procedures. On the other hand, the efficacy, excellence, or technical quality (TQ) of aesthetic treatments can be measured using various tools and scales such as the Aesthetic Global Ranking Scale, The Wrinkle Severity Rating Scale, and The Facial Laxity Rating Scale, to name but a few.^1-3^ However, it is unclear whether technical improvement, as rated by an assessment tool or healthcare provider (HCP), indeed correlates with the patient’s perception of quality or an optimal outcome.

Quality assessment comprises 2 distinct components: technical quality (TQ) and functional quality (FQ).^4^ On the one hand, TQ describes accuracy, excellence, and the degree to which procedures were performed “correctly.” In surgery, for example, TQ metrics would include successful treatment or cure of the presenting complaint; speed of return to normal function; and absence of morbidity, mortality, and complications. On the other hand, FQ embodies the way services are delivered and represents how the customer perceived and experienced the treatment or service. FQ comprises responsiveness, empathy, ease of access, and surroundings in healthcare.^5^ Factors such as communication time, intimacy of communication, and richness of information exchanged will influence FQ. In addition, patients’ psychological responses and behavior can be easily influenced by functional aspects of their care.^6^

Undoubtedly, most HCPs highly value TQ. Top healthcare outcome measures include mortality rate, readmission rate, safety, and effectiveness of care—all measures of TQ.^7^ Patients, however, typically have insufficient knowledge to accurately review TQ and, therefore, use substitute FQ to rate the overall quality of their care.^8^ Not even the highest levels of technical medical care can overcome shortfalls in the provision of the service.^9^ As aptly stated by Ware and Snyder, “patients cannot distinguish between the ‘caring’ performance and the ‘curing’ performance of medical care providers.” ^10^

Although it is understood that patient satisfaction is the predominant factor in determining the “success” of a cosmetic intervention,^11^ HCPs and academics within aesthetic medicine continue to center their research, education, and training around excellence in TQ. Anatomy, the aging process, facial assessment, safety, and avoidance of complications are at the forefront of aesthetic publications and education, in addition to a plethora of opinions on technical injection techniques.

Despite the emphasis on TQ in aesthetic medicine, it remains hugely subjective and difficult to assess. Cosmetic injectable practitioners frequently use standardized clinical photography to validate their TQ by displaying visible augmentation, reduced signs of aging, and altered aesthetic or a reduction in negative messages and nonverbal communication. However, in many cases, truly excellent cosmetic outcomes may be difficult to demonstrate in photography, despite being appreciated by the patient and practitioner. Although complication rates and undesirable cosmetic outcomes are also metrics of TQ, these are not always reported; furthermore, the treating HCP may be unaware of adverse outcomes due to patients seeking advice or further treatment from a new practitioner.

As seen in other areas of medicine, cosmetic injectable patients are unlikely to have sufficient knowledge to accurately assess or rate their procedure’s technical excellence and are likely to use alternative measures, namely FQ, to infer the quality of their results. The aesthetic patient’s inability to judge the technical result is compounded by innate negativity bias as well as possible self-esteem and confidence issues. Additionally, subjective aesthetic ideals and the subtle nature of some desirable treatments complicate assessment and consensus around optimal TQ, even among experts. It is hypothesized that both patient satisfaction and their assessment of TQ correlate strongly with their level of trust in the treating practitioner, representing a marker of FQ. Within the cosmetic injectable literature, relatively little attention has been given to either FQ or the factors impacting patient satisfaction.

This study was designed to explore reasons for return patronage, a clear indication of patient satisfaction, and the relationship between patient-assessed cosmetic outcomes and trust level in their practitioner. Although trust is difficult to measure instrumentally, individuals can easily self-assess and report their trust levels in others. Additionally, trustworthiness is dependent on FQ factors empathy, connection, and communication, and accordingly, trust levels can be used as an indicator of FQ.^5,12-15^ It was hypothesized that patient assessment of TQ (satisfaction with results) would correlate highly with trust levels in the injectable practitioner.

## METHODS

The Cosmetic Injectables Patient Experience Exploratory Study (CIPEES) was developed to explore patient motivation, mindset, engagement, and all aspects of the patient-practitioner relationship (Appendix). The survey, open to any person who had previously undergone cosmetic injectable treatments, was anonymous and completed online through snowball recruitment. The snowball approach uses a collaborative network to acquire data from a large study population.^16^ The survey was in the English language but open to participants globally. The survey was hosted on SurveyMonkey (San Mateo, CA) and was open for 10 months from September 2020 to June 2021. The study was approved by The St Vincent’s Hospital Melbourne (Victoria, Australia) Human Research Ethics Committee. There were no incentives offered or any paid advertisement. Written consent was provided, by which the participants agreed to the use and analysis of their data.

Respondents were asked to consider the reasons for choosing to return to a cosmetic injector for repeat treatment. The question was worded as follows, with an individually randomized factor sequence for each respondent: Thinking specifically about the reasons you would return to a specific cosmetic injectable practitioner, please RANK the following based on their importance to you, where 1 is most important and 5 is least important:

1.  Convenience or cost of treatment
2.  Customer service and experience
3.  Cosmetic result/outcome from the previous treatment/s
4.  Trust in my practitioner’s actions and ability
5.  Connection and understanding with my practitioner

Further questions sought to explore the correlation between patient-assessed TQ (patient satisfaction with the result of their cosmetic treatment) and FQ of the care provided, using the level of trust in the practitioner as a marker for FQ. The questions were worded as follows:

• Question

Please rate your overall feeling of satisfaction with the cosmetic results from your most recent injectable practitioner, where 0 is the worst cosmetic result/s possible and 10 is the best result/s possible.

• Question

Please rate your level of trust in your most recent injectable practitioner, where trust is your confidence that their treatments and actions are always in your best interest. Select level from 0, which is no trust at all, to 10 which is complete trust.

## RESULTS

Of the 1430 participants in the CIPEES survey, 95.6% identified as female and 4.4% male, with ages ranging from 18 years to >65 years (median 33 years old). Approximately, 66% completed all 15 questions in the survey. The respondents were made up of residents from 74 countries with 59.0% living in Australia, 10.0% the United States, 6.2% the United Kingdom, and small numbers across 71 other countries (Supplemental Table). The numbers were insufficient to analyze the differences between countries and cultures.

### Factors in Return Patronage

The question was completed by 1119 participants, with results presented in Table 1. The number one ranked reason chosen for returning to a previous cosmetic injector (return patronage) was “Trust in my practitioner’s action and ability,” with a relative weighting of 4.19. This was followed closely by “Cosmetic result/outcome from previous treatment/s,” with a relative weighting of 3.95. The third most important reason for return patronage was “Connection and understanding with my practitioner,” with a relative weighting of 2.83. “Customer service and experience” and “Convenience or cost of treatment” were ranked fourth and fifth, respectively, with a weighting of 2.10 and 1.94, respectively. The ranking of “reasons for return to the previous injector” did not vary across age groups, with little variation in relative weighting.

**Table 1.** Ranking and Relative Weighting of Reasons for Returning to the Previous Cosmetic Injectable Practitioner

Reason for returning to the previous cosmetic injectable practitioner	Overall ranking (n = 1120)	Relative weighting score	Age 18-34 y (n =4 63)	Relative weighting score	Age 35-54 y (n = 556)	Relative weighting score	Age 55-65+ y (n = 101)	Relative weighting score
Trust in my practitioner’s actions and ability	1	4.19	1	4.14	1	4.21	1	4.32
Cosmetic result/ outcome from previous treatment/s	2	3.95	2	4.01	2	3.93	2	3.77
Connection and understanding with my practitioner	3	2.83	3	2.79	3	2.82	3	3.06
Customer service and experience	4	2.1	4	2.13	4	2.07	4	2.08
Convenience or cost of treatment	5	1.94	5	1.93	5	1.97	5	1.77

### Relationship Between Patient-Rated Cosmetic Results and Trust

**Figure 1.**
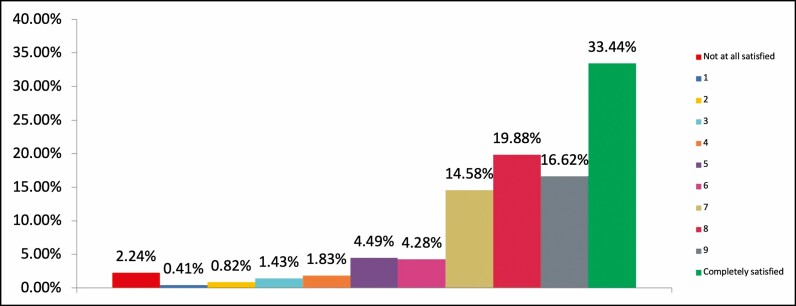
Satisfaction level. Chart showing responses to question: Please rate your overall feeling of satisfaction with the cosmetic results from your most recent injectable practitioner, where 0 is the worst cosmetic result/s possible and 10 is the best result/s possible (% of respondents, n = 981).

**Figure 2.**
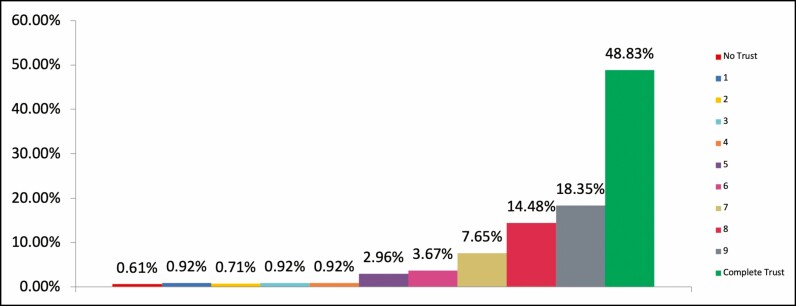
Trust level. Chart showing responses to question: Please rate your level of trust in your most recent injectable practitioner, where trust represents your confidence that their treatments and actions are always in your best interest. Select level from 0, which is no trust at all, to 10 which is complete trust.

The question asked respondents to rate their overall feeling of satisfaction with the cosmetic results, and their level of trust in the injector from their most recent injectable practitioner. Nine hundred eighty-one respondents completed both questions, and overall, there was both a high satisfaction level with their cosmetic results and level of trust in their injector (Figures 1, 2).

**Figure 3.**
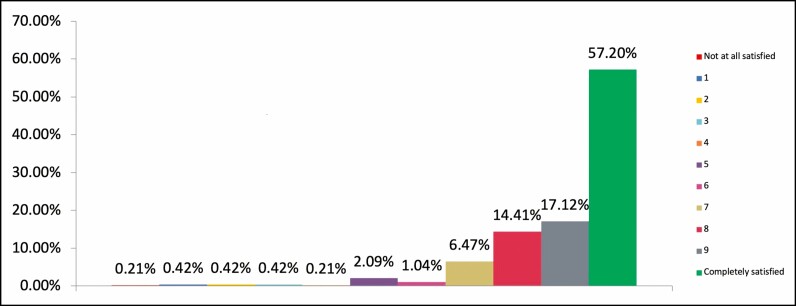
Chart showing overall feeling of satisfaction with the cosmetic results from most recent injectable practitioner for respondents who rated level of trust in practitioner as high as possible, 10/10 (% rated, n = 478).

**Figure 4.**
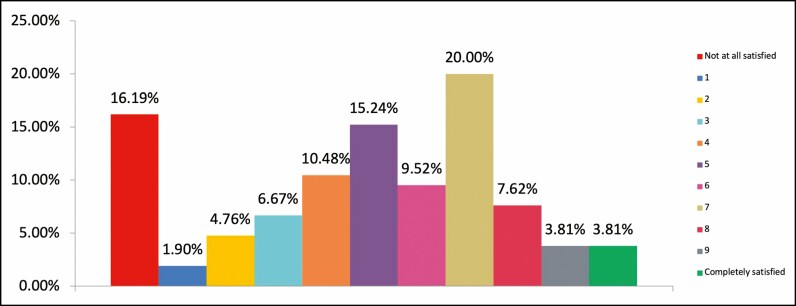
Chart showing overall feeling of satisfaction with the cosmetic results from most recent injectable practitioner for respondents who rated level of trust in practitioner as nil to moderate, 0/10–6/10 (% rated, n = 105).

Of the 478 respondents who rated their trust in their practitioner as 10/10 (complete trust), 88.7% were highly satisfied with their cosmetic result, rating it 8/10 or higher (Figure 3). Of the respondents (n = 105) demonstrating low to moderate trust levels in their practitioner (6/10 or less), only 15.2% had a high level of satisfaction with their cosmetic results (8/10 or higher) (Figure 4).

## DISCUSSION

Looking at return patronage and patient satisfaction, the findings confirm that “trust in the practitioner” is at least as important to patients as their technical results. Although trustworthiness increases with both TQ and FQ, and requires credibility, reliability, and intimacy, expertise alone (TQ) is insufficient to develop trust.^15,17^ Thus, we can extrapolate that functional care factors, such as empathy, connection, and communication, are very important to patients in feeling satisfied with their injectable treatments and in the decision to return to their cosmetic injectable practitioner.

Patient satisfaction is the ultimate goal in any aesthetic intervention. Cosmetic injectable HCPs generally attempt to improve patient satisfaction by becoming “better injectors” with a focus on TQ, aiming to avoid complications, improve injection skills, and achieve superior cosmetic outcomes. Although TQ education and training remain critical to safety and acceptable results, it needs to be understood that patients judge the overall quality of their result by both TQ and FQ, with FQ more important in determining patient satisfaction. High patient satisfaction leads to increased patient engagement behaviors that will promote better health outcomes and reduced risk.^18^ In aesthetic medicine, high patient engagement leads to adherence to treatment plans, positive reviews, word-of-mouth referrals, return patronage, and ultimately the long-term success of a medical aesthetic practice. Unsurprisingly, high patient satisfaction also leads to higher levels of practitioner job satisfaction.^19^

Specific research elucidating FQ within the field of aesthetic medicine and cosmetic injectables is limited but can be extrapolated from other fields. FQ can be broadly divided into practitioner and practice factors, with practice factors including ease of access, comfort of the physical surroundings, courteous front desk service, and administration staff and cost. Practitioner factors include connection, communication, honesty, attention, confidentiality, responsiveness, time spent in consultation, listening, patient education, and empathy, as seen in each patient interaction. As discussed earlier, these are also the factors involved in building trust.

Aesthetic medicine inhabits a precipitous divide between cut-throat commercialism and the benevolence of therapeutic medicine. It is further complicated by the delicate balance between mental health, societal pressures, empowerment and self-worth, and the desire for caring, empathy, and humanness. Despite a consumer mindset and high expectations influenced by social media, advertisements, and exaggerated claims in the public space, aesthetic patients still adopt FQ as a method of assessing overall treatment success and quality of care. Insufficient knowledge to judge TQ, and coupled with innate vulnerability, leads to increased importance of FQ aspects in the practitioner care of cosmetic injectable patients.

A study by Chung et al in a general plastic surgery clinic found 4 statistically significant factors predictive of patient satisfaction:^20^ (1) the personal manner of the physician, (2) the time spent with the physician, (3) length of time to get an appointment, and (4) a satisfactory explanation of what was done. Interestingly, 3 of the 4, other than time to get an appointment, are practitioner FQ factors. Point 4 (Satisfactory explanation of what was done) highlights the importance of patient education as a key factor in patient satisfaction.^21^ While patients are currently typically more educated before attending an aesthetic consultation, it is erroneous to assume that they understand the objectives, risk, or expected outcome of any planned procedure. FQ factors such as time spent with the HCP, empathy, and communication are consistently found to be key contributors to patient satisfaction.^22-25^ Communication in aesthetic practice is becoming an autonomous academic discipline, requiring assertiveness, empathy, and shared decision making.^26-28^

The authors acknowledge the limitations of this study, particularly with regard to the inherent selection and response bias with an online survey format, and the vastly heterogenous group of participants. Ideally, prospective qualitative research and case-controlled studies would more accurately elucidate the true role of functional quality in patient satisfaction.

## CONCLUSIONS

Patient loyalty is highly sought after in the aesthetic industry, representing a marker of patient satisfaction and leading to an enjoyable and sustainable career for the practitioner. While business minds may elect advertising, marketing, packages, and discounts to encourage patronage, HCP focus should remain on qualifications, training, and technical expertise. Patients, however, tend to value functional quality more highly, reflecting a measure of how their care was delivered. Furthermore, it has been shown that when asked to assess the TQ of their procedure, patients may instead infer the TQ from their judgment of FQ instead.

In this study, trust in the cosmetic injectable practitioner was reported as the number one reason for return patronage, and satisfaction with the cosmetic result correlated highly with the level of trust in the practitioner. Interestingly, this did not fluctuate with age or generation. It seems that caring (humanness) could be more important than curing (competence). In order to maximize patient satisfaction and patient loyalty, HCPs should thus focus on improving FQ and value it at least as highly as TQ in their delivery of cosmetic injectables.

## Supplementary Material

ojac044_suppl_Supplementary_Material_S1

ojac044_suppl_Supplementary_Material_S2
